# Aberrant activation of NF-κB signaling in mammary epithelium leads to abnormal growth and ductal carcinoma in situ

**DOI:** 10.1186/s12885-015-1652-8

**Published:** 2015-09-30

**Authors:** Whitney Barham, Lianyi Chen, Oleg Tikhomirov, Halina Onishko, Linda Gleaves, Thomas P. Stricker, Timothy S. Blackwell, Fiona E. Yull

**Affiliations:** 1 Department of Cancer Biology, Vanderbilt University Medical Center, 23rd Ave S and Pierce PRB 325, Nashville, TN 37232 USA; 2 Department of Medicine, Division of Allergy, Pulmonary and Critical Care Medicine, Vanderbilt University Medical Center, 1161 21st Ave, Nashville, TN 37232 USA; 3 Department of Pathology, Vanderbilt University Medical Center, 1161 21st Ave, Nashville, TN 37232 USA; 4 Vanderbilt-Ingram Cancer Center, 691 Preston Building, 2220 Pierce Ave, Nashville, TN 37232 USA

**Keywords:** Nuclear factor kappa-B, Mammary, Inflammation, Hyperplasia, Ductal carcinoma in situ, Mucin 1

## Abstract

**Background:**

Approximately 1 in 5 women diagnosed with breast cancer are considered to have *in situ* disease, most often termed ductal carcinoma in situ (DCIS). Though recognized as a risk factor for the development of more invasive cancer, it remains unclear what factors contribute to DCIS development. It has been shown that inflammation contributes to the progression of a variety of tumor types, and nuclear factor kappa B (NF-κB) is recognized as a master-regulator of inflammatory signaling. However, the contributions of NF-κB signaling to tumor initiation are less well understood. Aberrant up-regulation of NF-κB activity, either systemically or locally within the breast, could occur due to a variety of commonly experienced stimuli such as acute infection, obesity, or psychological stress. In this study, we seek to determine if activation of NF-κB in mammary epithelium could play a role in the formation of hyperplastic ductal lesions.

**Methods:**

Our studies utilize a doxycycline-inducible transgenic mouse model in which constitutively active IKKβ is expressed specifically in mammary epithelium. All previously published models of NF-κB modulation in the virgin mammary gland have been constitutive models, with transgene or knock-out present throughout the life and development of the animal. For the first time, we will induce activation at later time points *after* normal ducts have formed, thus being able to determine if NF-κB activation can promote pre-malignant changes in previously normal mammary epithelium.

**Results:**

We found that even a short pulse of NF-κB activation could induce profound remodeling of mammary ductal structures. Short-term activation created hyperproliferative, enlarged ducts with filled lumens. Increased expression of inflammatory markers was concurrent with the down-regulation of hormone receptors and markers of epithelial differentiation. Furthermore, the oncoprotein mucin 1, known to be up-regulated in human and mouse DCIS, was over-expressed and mislocalized in the activated ductal tissue.

**Conclusions:**

These results indicate that aberrant NF-κB activation within mammary epithelium can lead to molecular and morphological changes consistent with the earliest stages of breast cancer. Thus, inhibition of NF-κB signaling following acute inflammation or the initial signs of hyperplastic ductal growth could represent an important opportunity for breast cancer prevention.

**Electronic supplementary material:**

The online version of this article (doi:10.1186/s12885-015-1652-8) contains supplementary material, which is available to authorized users.

## Background

How cancer starts is a topic of considerable debate. In the case of breast cancer, many believe that changes in the ductal or lobular epithelium begin subtly and then progress along a continuum until they become malignant and eventually metastatic [1]. Mirroring this progression are changes in the architecture and structure of the ductal epithelium: an organized bilayer of cells begins to exhibit atypia, hyperplasia, ductal occlusion, and eventually advances to a chaotic mass [2]. This implies that finding the earliest abnormalities in ductal structure will help the clinician to intervene before the accumulated effects become life-threatening. It is based on this assumption that thousands of women are encouraged to undergo mammograms each year, and a subset to undergo tissue biopsy as a result of detection of radiographic abnormalities.

With an increased prevalence of screening, there has also been an increase in the detection of early stage lesions, many termed “ductal carcinoma in situ” (DCIS) [3]. DCIS is considered one of the earliest forms of breast cancer and is characterized by proliferating ductal epithelial cells exhibiting atypia, but not yet breaking through the basement membrane. Approximately 20 % of all breast cancer diagnoses in the United States (about 60,000 cases per year) are deemed *in situ* [4]. The presence of these early lesions within the breast is recognized as a risk factor for invasive breast cancer occurrence, so women are treated with aggressive therapy such as lumpectomy or mastectomy sometimes followed by radiation [5]. However, the field has yet to truly understand the natural history of DCIS [6]. It remains unclear what factors contribute to its development and progression. If these factors could be determined, could we inhibit them and prevent hyperplastic lesions from occurring? In addition, are there specific signaling pathways that could be blocked to prevent them from progressing? These are critical questions, the answers to which would affect thousands of women each year.

Inflammation is recognized as a critical component for the progression of a variety of cancers [7]. Nuclear Factor Kappa-B (NF-κB) is a family of transcription factors that regulate inflammatory signaling. The most widely-studied members of this family are part of the canonical pathway, where upstream signaling induces phosphorylation of the Inhibitor of Kappa-B kinase-beta (IKKβ). This in turn phosphorylates the Inhibitor of Kappa B alpha (IκBα), targeting it for degradation. With the inhibitor gone, p65/p50 heterodimers once held in the cytoplasm are free to enter the nucleus and affect transcription of downstream gene targets [8–11]. These include genes that participate in a wide range of cellular processes such as proliferation, apoptosis, angiogenesis, and cytokine release. It has been shown that NF-κB activity within breast tissue can increase due to stimuli such as obesity, acute infection, or physiological stress [12–14].

In a previous mammary development study, Brantley et. al found that IκBα knock out (KO) transgenic mouse epithelium develops abnormally, with hyper-branched structures and filled ductal lumens [15]. This was the first hint that there might be a link between NF-κB activation and the initiation of aberrant growth in breast epithelium. Though we and others have previously drawn a connection between NF-κB activation and mammary tumor *progression,* these experiments were all performed in combination with strong oncogenic or carcinogenic tumor models [16–19]. In contrast, the study noted above attempted to model the consequences of NF-κB activation within developing breast epithelium in the absence of any other tumorigenic stimuli.

In the current work, we use a novel doxycycline (dox) inducible transgenic mouse model to acquire deeper insights into whether activated NF-κB signaling in the mammary epithelium could play a role in the formation of hyperplastic breast lesions. In these transgenics, NF-κB is activated through expression of a constitutively active IKKβ (cIKKβ) in mammary epithelial cells [12]. Our system not only directs activation to a specific cell type (mammary epithelium), but it allows temporal control of that activation. All previously published models of NF-κB modulation to investigate development of the virgin mammary gland have been constitutive models, with transgene or KO present throughout the life and development of the animal. For the first time, we can induce activation at later time points *after* normal ducts have formed, thus being able to determine if NF-κB activation can promote pre-malignant changes in previously normal mammary epithelium. Through these studies, we show that NF-κB activation in the virgin mammary gland can lead to rapid molecular and morphological changes consistent with early mammary tumorigenesis, including hyperproliferation of ductal epithelial cells, filling of ductal lumens, macrophage infiltration, and increased expression and mislocalization of the oncogene Mucin 1 (MUC1).

## Methods

### IKMV mouse model

All animal experiments were approved by the Vanderbilt University Institutional Animal Care and Use Committee. Transgenic mice containing the NF-κB activating (tet-O)7-FLAG-cIKK2 construct [20] were mated with mouse mammary tumor virus-reverse tetracycline transactivator (MMTV-rtTA) mice [21] (gift from Dr. L. Chodosh, School of Medicine, University of Pennsylvania, Philadelphia, PA). This cross produced pups carrying both transgenes which were designated IKMV, as previously described [12]. Littermates lacking one or both transgenes were used as controls. All mice were on an FVB strain background. IKMV females (or littermate controls) were maintained on normal water until transgene activation was required. At the appropriate experimental time point, both IKMV and control virgin females were treated with freshly prepared doxycycline (dox) (Sigma-Aldrich), given *ad libitum* in drinking water (1 – 2 g/L). Sucrose (5 %) was also added to decrease the bitter taste of dox water. A red bottle was used to prevent light-induced dox degradation and water was replaced twice per week.

### TransAM ELISA

Nuclear extracts from whole mammary tissue were obtained using our previously described methods [22]. Halt protease/phosphatase inhibitor cocktails (Pierce) were added to lysis buffers. Following extraction, protein concentration was assessed using a Bradford assay (BioRad). TransAM ELISA (Active Motif) was completed according to manufacturer’s instructions using the anti-p65 antibody provided in the NF-κB family member kit (Cat #43296). 8 micrograms of nuclear extract were added to each well, and samples were run in duplicate. A total of 4 control samples and 4 IKMV samples (6 week virgin, 3 days dox treated time point) were compared for the graph and statistics.

### Mammary gland transplant

General procedures for isolation and transplantation of mammary epithelial tissue have been demonstrated previously [23]. Details of our protocol were also described in a previous manuscript [15]. With regard to the current studies, IKMV donor mammary tissue from 3–4 week old donors was transplanted into the cleared fat pad of the left inguinal mammary gland of 3 week old FVB wild type recipient females. Donor tissue taken from a littermate control was transplanted into the contralateral cleared fat pad. Tissue was collected and transplanted on the same day (no cryopreservation). After transplant, recipient mice were monitored through a 3 day recovery period during which they remained on normal water. 72 h post-transplant, the mice began dox treatment (2 g/L), which was continuous until sacrifice. The mammary glands were analyzed 3 or 4 weeks after transplantation.

### Mammary whole mount staining

Number 4 inguinal mammary glands of dox-treated mice were collected and spread on microscope slides at the time of sacrifice. Glands were then fixed overnight in formalin at 4 °C followed by haematoxylin staining as previously described [22]. Images were captured using a dissecting microscope and Canon Powershot A590 camera. If mice underwent transplant, the fat pad in which the transplanted tissue had been inserted was collected and placed on a microscope slide. This was then prepared and imaged in the same way as the intact IKMV and control glands.

### TEB size quantification

Whole mount images were analyzed using MetaMorph software (Molecular Devices). A photo was taken of a standard ruler at the time the whole mount images were captured, using identical parameters and magnification. After images were loaded into MetaMorph, a circle was drawn around the TEB. Using the ruler photo for calibration, the software translated each region into an area measurement. The same calibration was applied to all images analyzed. 5 control and 6 IKMV transplanted glands were used for comparison of TEB size. 5 TEB’s from each whole mount were measured and values averaged.

### Branching quantification

Branching was quantified using Photoshop CS4 software (Adobe). Whole mounts of IKMV and control transplanted glands, treated with dox for 3 weeks, were imaged at the same session and using the same magnification. Photos were then loaded into the program and a grid of 75 mm squares was digitally overlaid onto each image. The number of bifurcations observed in each square was manually counted. At least 8 individual squares were counted per gland and the values averaged. 4 separate control transplanted glands and 4 IKMV transplanted glands were compared for quantification.

### Histology (H&E’s)

Number 4 inguinal mammary glands (intact or transplanted fat pads) were fixed in 10 % formalin overnight at 4 °C. The glands were then dehydrated in a graded ethanol series followed by xylenes and embedded in paraffin. 5 μm sections were prepared and stained with haematoxylin and eosin (Vanderbilt University Medical Center, Allergy, Pulmonary, and Critical Care Medicine Immunohistochemistry Core).

### Area of duct quantification

To quantify the area of each duct, H&E stained slides were used. Terminal end buds (found at the leading edge of the 6 week old glands) were excluded from all analyses. Images of ducts were captured using a Zeis Axioplain 2 microscope at 20X magnification. After capture, images were analyzed using MetaMorph software (Molecular Devices). The outer edge of each duct was traced using the drawing feature to form a polygon. The area of the polygon was then determined based on a calibration scheme (pixels to micrometers) previously performed by the Cancer Biology Microscopy Core using the 20X objective and MetaMorph software. This resulted in an area measurement for each duct in micrometers squared. If a lumen was present in the duct, the edges of the lumen were traced to form a second polygon. This area measurement was subtracted from the first to yield the area actually containing cells in each duct. 3 control glands and 3 IKMV glands from the each time point (6 week virgin or 16 week virgin) were analyzed. A minimum of 8 ducts per gland were measured.

### Immunohistochemistry

PCNA staining was completed using formalin fixed, paraffin embedded tissue. Slides were deparaffinized using xylenes and a graded ethanol series and antigen retrieval was completed using citrate buffer (pH 6) and steam heat. After blocking with 1 % BSA, slides were incubated with Biotin-conjugated PCNA monoclonal antibody (Life Technologies) at a 1:100 dilution for 1.5 h at room temperature. VECTASTAIN Elite ABC Kit (Mouse IgG) and VECTOR NovaRED Peroxidase (HRP) Substrate Kit were used for visualization (Vector Laboratories, Inc.), and slides were counterstained with haematoxylin. Images of 6 ducts per slide were captured using a Zeis Axioplain 2 microscope at 20X magnification. Images were then loaded into MetaMorph software (Molecular Devices) for quantification. Positive cells were manually counted and the number of positive cells normalized to the total area of each duct (area calculated as described above). Mammary glands from 3 control and 3 IKMV glands were used for quantification and 6 ducts per gland were counted. TEB’s were excluded from all analyses. F4/80 staining was completed by the Vanderbilt Translational Pathology Shared Resource using a rat anti-mouse monocolonal antibody against F4/80 (CI:A3-1) (Novus Biologicals). Images were captured using a Zeis Axioplain 2 microscope at 20X magnification.

Immunofluorescent staining was completed using formalin fixed, paraffin embedded mammary tissue sections and primary antibodies against: MUC1 (AbCam); Cytokeratin-5 (Covance); Cytokeratins 8/18 (RDI-Fitzgerald); Smooth muscle actin (SMA) (CalBiochem); FLAG (Sigma); Ki-67 (Abcam); ERα (Thermo Fisher); and phospho-p65 (ser536) (Cell Signaling). The staining protocol was similar to above, but required blocking with 2 % BSA and goat serum, and addition of appropriate secondary antibodies tagged with either Alexa Fluor 488 or Alexa Fluor 594 (both Life Technologies). Slides were coverslipped using Molecular Probes ProlongGold antifade reagent (Life Technologies) to preserve fluorescence. Images were then captured using either a Zeis Axioplain 2 microscope or a LSM 510 Meta confocal microscope in the Vanderbilt University Medical Center Imaging Core. Either TO-PRO-3 (Life Technologies) or DAPI (Sigma) were used as nuclear stains.

### Flow cytometry

Following sacrifice, mammary glands #2-4 were harvested for analysis. Lymph nodes of the #4 glands were removed prior to collection. Glands were minced and placed in 3 mL’s of DMEM/F12 containing 3 mg/mL of Collagenase A (Roche) and 100 units/mL Hyaluronidase (Sigma). Glands were incubated in digestion media overnight at 4 °C, followed by 2 h of incubation at 37 °C the following morning. After digestion, cells were pelleted and the fatty layer at the top of the supernatant was discarded. After straining cells through a 70 micron filter, red blood cells were lysed using ACK buffer. Remaining cells were then washed and counted using a hemocytometer. Cells were blocked with anti- mouse CD16/CD32 antibody (eBioscience) before staining with anti-mouse antibodies: CD45 (30-F11) (eBioscience) and F4/80 (BM8) (Life Technologies). DAPI nuclear stain was used to determine viability. Analysis was performed on an LSRII cytometer with DIVA software (BD Biosciences). Gating strategy can be found in Additional file 1. Values for the graph in Fig. 7b were obtained by taking the total number of CD45^+^F4/80^+^ positive cells for each sample and dividing that value by the total number of viable cells in the sample (DAPI negative).

### RNA isolation and RT-PCR

Mammary gland total RNA was extracted using Trizol (Invitrogen) and the RNeasy Mini Kit (Qiagen), as previously described [12]. RT-PCR was utilized to detect expression of the FLAG-tagged cIKKβ transgene (annealing temperature of 58 °C and a 35 cycle program). For all other gene targets, qRT-PCR was performed using the Applied Biosystems Stepone Plus Real-Time PCR system and SYBR Green PCR Master Mix (Applied Biosystems) (annealing temperature of 60 °C and a 40 cycle program). All primer sequences used are contained in Table 1. Each primer pair was tested and melt curves analyzed to ensure that only a single amplicon was generated. All experimental and control samples were assayed in triplicate for target gene or GAPDH (reference gene). The average of the three CT values was used as “CT” for each sample. For graphical representation, target gene CT values (A) and GAPDH CT values (B) were both expressed as exponents of 2, and data represented as the ratio of 2A/2B, or 2^(A - B)^. The exception is Fig. 7a, which contains qRT-PCR data graphed as log fold change. These values were produced using the 2^-Δ(ΔCT)^ comparative method [24] and then GraphPad Prism software was used to put those values on a log scale. P values for the statistical comparison of the data in Fig. 7a are in Table 2.

**Table 1:** A comprehensive list of all real time primer sequences used in our studies

Gene	Forward (5'-3')	Rev (5'-3')
GAPDH	TGAGGACCAGGTTGTCTCCT	CCCTGTTGCTGTAGCCGTAT
CIKK2-FLAG	GGAGCTCCACCGCGGTGCGG	TCAGGGACATCTCGGGCAGC
Cyclin bl	AAGGTGCCTGTGTGTGAACC	GTCAGCCCCATCATCTGCG
pl8^INK4c^ (CDKN2C)	CCTTGGGGGAACGAGTTGG	AAATTGGGATTAGCACCTCTGAG
Muc-1	GGCATTCGGGCTCCTTTCTT	TGGAGTGGTAGTCGATGCTAAG
CXCL1	CCGAAGTCATAGCCACACTCAA	GCAGTCTGTCTTCTTTCTCCGTTAC
ILl-β	GCAACTGTTCCTGAACTCAACT	ATCTTTTGGGGTCCGTCAACT
TNF-α	AGGCACTCCCCCAAAAGATG	TCACCCCGAAGTTCAGTAGACAG A
Cox-2	CCAGCACTTCACCCATCAGTT	ACCCAGGTCCTCGCTTATGA
CCL-2	CCCACTCACCTGCTGCTACT	TCTGGACCCATTCCTTCTTG
IL-12	GGAAGCACGGCAGCAGAATA	AACTTGAGGGAGAAGTAGGAATGG
RANK	CCAGGAGAGGCATTATGAGCA	ACTGTCGGAGGTAGGAGTGC
ERα	CCTCCCGCCTTCTACAGGT	CACACGGCACAGTAGCGAG
Prolactin-R	CACTTGCTTACATGCTGCTTG	CAGGTGGTGACTGTCCATTCA
Progesterone-R	GACACTGGCTGTGGAATTTCC	CCAGGATCTTGGGCAACTG
Elf5	ATGTTGGACTCCGTAACCCAT	GCAGGGTAGTAGTCTTCATTGCT
Csn2	GGCACAGGTTGTTCAGGCTT	AAGGAAGGGTGCTACTTGCTG

**Table 2:** Statistical significance values for qRT-PCR shown as fold change in Fig. 7a

Gene	Samples Assayed (control, IKMV)	*p* value
CXCL1	*n* = 3, *n* = 4	0.0067
ILl-β	*n* = 6, *n* = 6	0.0241
TNF-α	*n* = 6, *n* = 7	0.0336
Cox-2	*n* = 6, *n* = 7	0.0593
CCL-2	*n* = 3, *n* = 3	0.0159
IL-12	*n* = 3, *n* = 3	0.0169
RANK	*n* = 5, *n* = 7	0.0100
ERα	*n* = 6, *n* = 7	0.0424
Prolactin-R	*n* = 6, *n* = 7	0.0100
Progesterone-R	*n* = 6, *n* = 7	0.0053
Elf 5	*n* = 6, *n* = 6	0.0031
Csn2	*n* = 4, *n* = 4	0.0038

### Data analysis

Statistical analyses were performed using GraphPad Prism (GraphPad Software Inc.). In each case, paired t-statistics with *p*-value < 0.05 were used to determine whether the values in IKMV tissue were significantly different from those in control. Data are plotted graphically as mean vertical bars representing standard error (except for Fig. 7a). The height of the bars in Fig. 7a represent average fold change, as described above, and do not contain standard error bars.

## Results

### IKMV transgenic mouse model targets expression of cIKKβ specifically to mammary epithelium

**Fig. 1:**
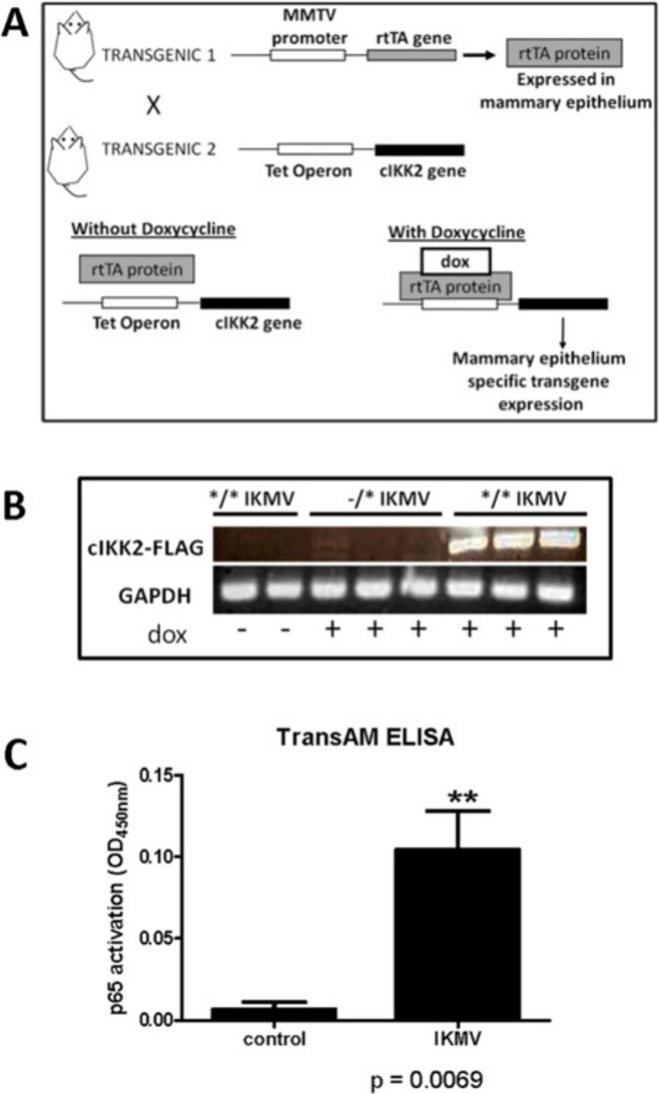
*Transgenic mouse model targets expression of cIKKβ specifically to mammary epithelium.* **a** Diagram shows crossing of two transgenic strains necessary to generate the double transgenic (*/*) IKMV mouse model with doxycycline inducible transgene expression. Littermates lacking either one or both transgenes (*/-, −/*, or −/−) were used throughout our studies as littermate controls. For characterization, IKMV and control littermates were treated with doxycycline (2 g/L) for 3 days and mammary tissue collected for the following assays: **b** RT-PCR of whole mammary homogenates confirms the FLAG-tagged transgene is dox-inducible. Upon dox-treatment, the transgene was expressed in the */* double transgenic IKMV animals, but absent in dox-treated, single transgenic control mice (−/*). Double transgenic */* IKMV mice that did *not* receive dox-treatment showed no detectable transgene expression. **c** TransAM ELISA assay using IKMV and control mammary nuclear homogenates shows that nuclear p65 in IKMV samples actively binds the NF-κB DNA consensus sequence (*n* = 4 control, *n* = 4 IKMV samples; **p = 0.0069)

Previously, our group has studied the activation of NF-κB in mammary tissue *in vivo* using IκBα knock-out mice [15]. In these transgenics, deletion of the inhibitor is systemic and activity through the canonical pathway is increased within every tissue, causing mortality by day 9 post birth [25]. However, transplant of mammary tissue from 6 day old female pups into wild type donors allowed us to observe the effects of NF-κB activation during pubertal mammary gland development. Using this model, we found an increase in lateral ductal branching and pervasive intraductal hyperplasia in the IκBα knock-out reconstituted glands. This was the first indication that aberrant NF-κB activation could lead to dramatic changes in ductal growth. As in most mammary transplant methods, stromal and epithelial components were co-transplanted into recipients. Because IκBα had been deleted in both of these components, it was impossible to determine whether it was the epithelial derived NF-κB activation that caused the resulting phenotype. To address this and to enable specific temporal regulation of the increased activation of NF-κB, we developed a doxycycline (dox) inducible model which would target activation specifically to mammary epithelium. This model requires two transgenic components: tet-O-cIKKβ mice are combined with MMTV-rtTA transgenics to produce double transgenic mice that we have termed “IKMV” (Fig. 1a). RT-PCR of whole mammary homogenates confirms the FLAG-tagged cIKKβ transgene is dox-inducible. Upon dox-treatment, transgene expression was evident in the */* double transgenic IKMV mammary, but absent in dox-treated, single transgenic control mice (−/*). Double transgenic */* IKMV mice that did *not* receive dox-treatment showed no detectable transgene expression (Fig. 1b). Thus, in all subsequent studies, “IKMV” refers to the double transgenic mice and “control” refers to littermates lacking one or both transgenes, which behave as wild type mice. To confirm the ability of the transgene to activate NF-κB activity, TransAM ELISA was completed using the nuclear fraction of mammary tissue lysates. This showed that there is increased binding of nuclear p65 to the NF-κB DNA consensus sequence following transgene induction (Fig. 1c).

### Mammary epithelial expression of cIKKβ during ductal development results in enlarged terminal end-buds, increased lateral branching, and hyperplasia

**Fig. 2:**
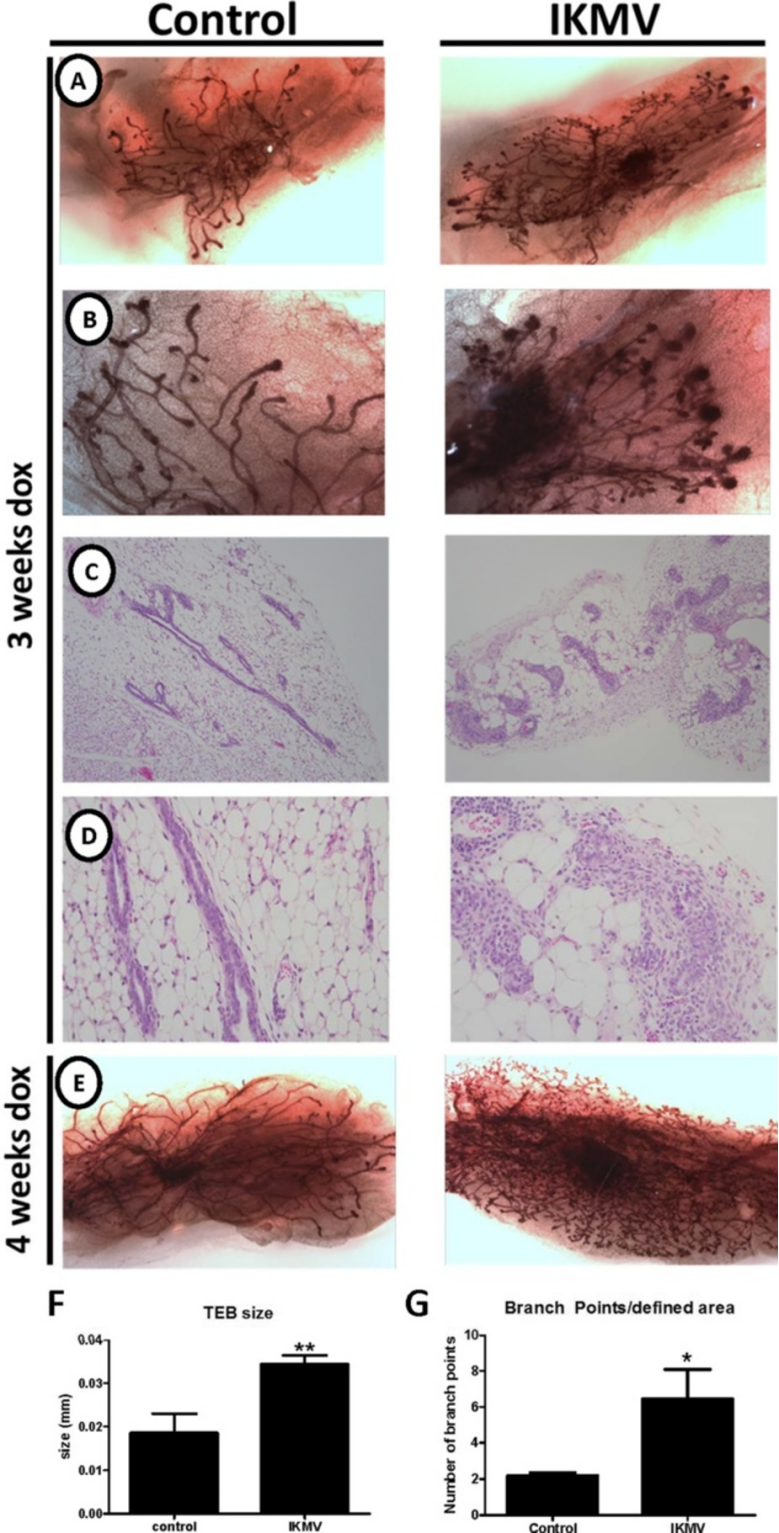
*Expression of cIKKβ in transplanted mammary epithelium results in intraductal hyperplasia.* Mammary tissue from 3–4 week old IKMV donors was transplanted into the cleared fat pad of the #4 mammary gland of 3 week old FVB recipients. Tissue from control littermates was transplanted into the contralateral #4 gland. After a 72 h recovery period, mice were placed on dox treatment (2 g/L), which was continuous until sacrifice at 3 or 4 weeks post-transplant. **a** Haematoxylin stained whole mounts of mammary fat pads after 3 weeks of growth in recipient mice reveal increased lateral branching of IKMV tissue. **b** Higher magnification highlights swollen end buds in IKMV. Hyperplasia of the IKMV ducts is evident in images of H&E stained tissue using **c** 10X and **d** 20X objectives. **e** Whole mounts of mammary tissue after 4 weeks of growth indicate that IKMV tissue continues to fill fat pad with hyperplastic tissue. The observed phenotype was quantitatively assessed through: **f** quantification of terminal end bud (TEB) size (*n* = 5 control, *n* = 6 IKMV glands, **p = 0.0071) **g** quantification of the number of lateral branch points per field (*n* = 4 control, *n* = 4 IKMV glands, *p = 0.0416)

After validating the IKMV transgenic system, we used the mammary transplant model to produce samples that could be directly compared to our studies using the IκBα KO mice. To do this, IKMV donor mammary tissue was transplanted into the cleared fat pad of 3 week old FVB wild type recipient females. Donor tissue taken from a littermate control lacking one or both transgenes was transplanted into the contralateral cleared fat pad. Recipient mice were dox-treated continuously following the transplant, and glands were analyzed at both 3 and 4 week post-transplant time points. Haematoxylin stained whole mounts of the tissue reveal that the IKMV ductal tree has on average three times the number of lateral branch points as the control transplants after 3 weeks of growth (Fig. 2). The IKMV ducts are not only hyper-branched, but also hyperplastic, as H&E staining clearly shows filled lumens and increased cell density throughout the transgenic ducts. In addition, we found that the terminal end-buds of the IKMV glands were larger than the controls. These studies definitively show that *epithelial* specific NF-κB activation during ductal branching morphogenesis results in abnormal branching and hyperplastic ductal growth.

### Short term activation of NF-κB results in dramatic morphological changes within previously normal mammary ductal structure

**Fig. 3:**
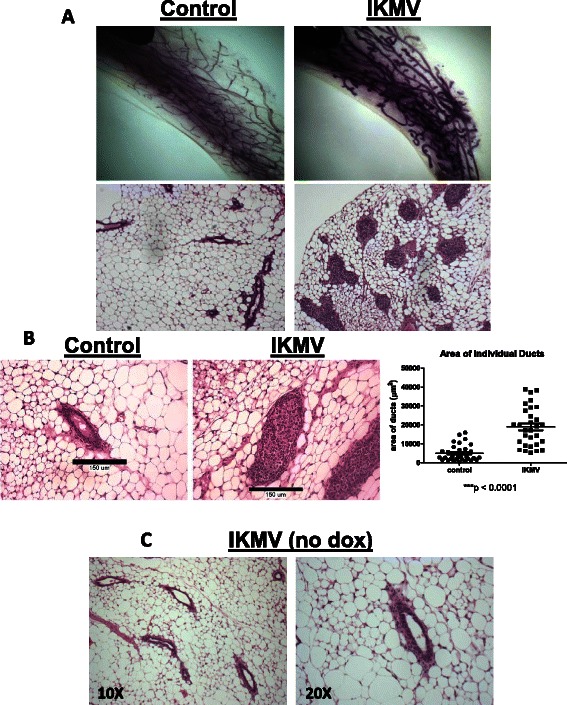
*Short term activation of NF-κB in mammary epithelium leads to ducts with filled lumens.* Intact 6 week old virgin IKMV and control littermates were dox-treated (2 g/L) for 3 days prior to sacrifice. **a** Haematoxylin stained whole mounts of control and IKMV glands reveal changes in IKMV ducts. In H&E stained sections (below), we observed a complete occlusion of IKMV ducts throughout the gland. **b** Increased size of individual IKMV ducts is apparent in 20X images with calibration bars (150 μm). Multiple measurements of duct area across samples are quantified at right (*n* = 3 control, *n* = 3 IKMV glands, total of 65 individual ducts were measured; *** *p* < 0.001). **c** Double transgenic IKMV virgin females were kept on normal water at the 6 week virgin time point and collected 3 days later along with the dox-treated cohort. Images of H&E stained mammary tissue show ducts of untreated controls have normal morphology, with no lumen-filling or hyperplastic growth (10X magnification at left, 20X at right)

In our transplant studies, outgrowth of the mammary ducts and NF-κB activation had been simultaneous, starting when the hosts were 3 weeks of age. In order to better model early tumorigenesis without the overlay of developmental abnormalities, we induced NF-κB signaling *after* a subset of normal ductal structures had already formed. To do this, we took 6 week old virgin, intact IKMV and control females and dox-treated them for 3 days prior to collection. Surprisingly, we found that after this short pulse of transgene induction, striking changes had occurred throughout the IKMV ductal structure. The lumens of the IKMV ducts were completely filled with cells and the ducts were significantly enlarged (Fig. 3a,b). This phenotype is fully penetrant and occurs in 100 % of the ducts throughout the IKMV glands. As an added control, non-dox treated, double transgenic IKMV females were collected at the same 6 week old, virgin time-point. Mammary tissue from these untreated controls appeared normal, with no lumen-filling or hyperplastic ducts (Fig. 3c). This confirmed that the phenotype in the dox-treated IKMV mice occurred within the 3 day induction window.

**Fig. 4:**
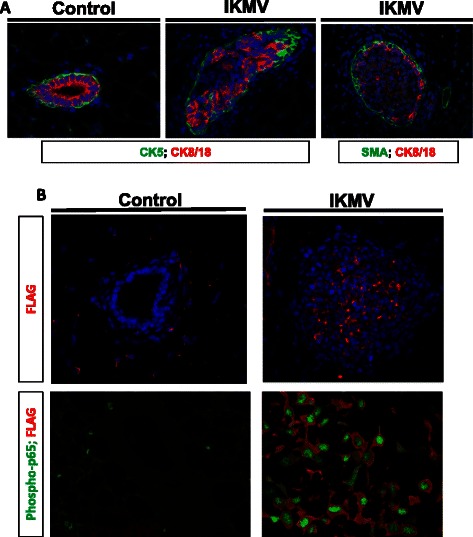
*Many cells within aberrant ducts are epithelial, transgene-expressing, and have high levels of NF-κB activation.* 6 week old virgin IKMV and control littermates were dox-treated (2 g/L) for 3 days prior to sacrifice. **a** Immunofluorescent staining of control and IKMV tissue reveals that IKMV ducts are filled with CK8/18 positive luminal epithelium. CK5 and SMA were used as markers of basal/myoepithelium. **b** Separate staining shows that the FLAG-tagged cIKKβ transgene is expressed by cells within the IKMV hyperplastic ducts (red, FLAG stain). In addition, high magnification images of ductal tissue from IKMV and control littermates confirmed that the transgene is localized appropriately within the cytoplasm of IKMV mammary epithelium and is driving concurrent nuclear localization of phospho-p65 (green)

Upon observing such a dramatic filling of the IKMV ducts, we endeavored to determine whether the cells within the ducts were epithelial. To do this, we completed immunofluorescent staining for the luminal epithelial marker cytokeratin 8/18 (CK8/18). This revealed that many of the cells filling the lumens stain positive for this marker (Fig. 4a). In addition, FLAG-tagged transgene expression was found throughout the aberrant ducts in IKMV glands (Fig. 4b). Since transgene expression is specific to MMTV-rtTA expressing mammary epithelial cells in the IKMV system, this again suggests that many of the cells filling the ducts are epithelial. Finally, we wanted to confirm that the transgene expression was indeed driving NF-κB activation within the epithelium at the 3 day time point. Using immunofluorescent staining, and high magnification images, we observed cytoplasmic localization of the transgene within mammary epithelial cells resulting in robust nuclear localization of phospho-p65 (ser 536) (Fig. 4b).

**Fig. 5:**
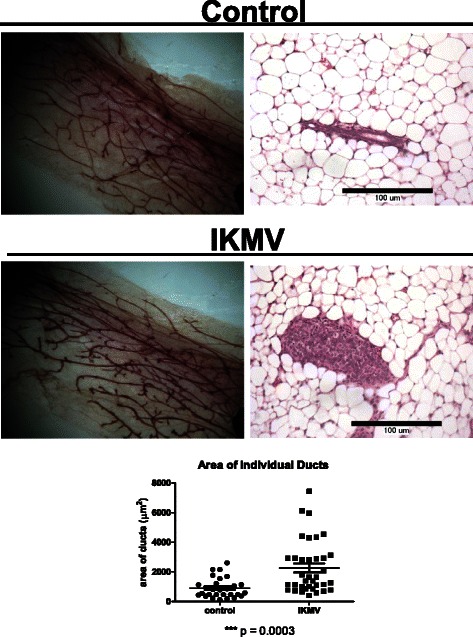
*Abnormal ducts induced in fully adult, virgin glands through activation of NF-κB in mammary epithelium.* 16 week old adult, virgin IKMV and control females, which were previously untreated, were given dox (1 g/L) for 10 days prior to sacrifice. A subtle enlargement of ducts can be seen in haematoxylin stained whole mounts (left panels). The phenotype is more apparent in H&E stained sections (100 μm calibration bar) (right panels). IKMV ducts are filled with cells and significantly larger than the controls. Size of ducts is quantified below images (*n* = 3 control and *n* = 3 IKMV glands, total of 64 ducts were measured; ***p = 0.0003)

As 6 week old virgin mice are still undergoing puberty, the mammary tissue may be responding to a higher level of hormonal stimulation than quiescent, adult glands. To determine if the phenotype would also occur in adult mice, we treated 16 week old virgin IKMV and control females with dox for 10 days. Upon collection, we saw that the IKMV ducts were significantly larger than the control ducts in cross sectional area and had indeed become filled with cells (Fig. 5). This indicated that the notable changes in the IKMV ductal structure after a short-term induction of NF-κB activity were not dependent on puberty-related physiological factors.

### Cells filling the abnormal ducts are highly proliferative

**Fig. 6:**
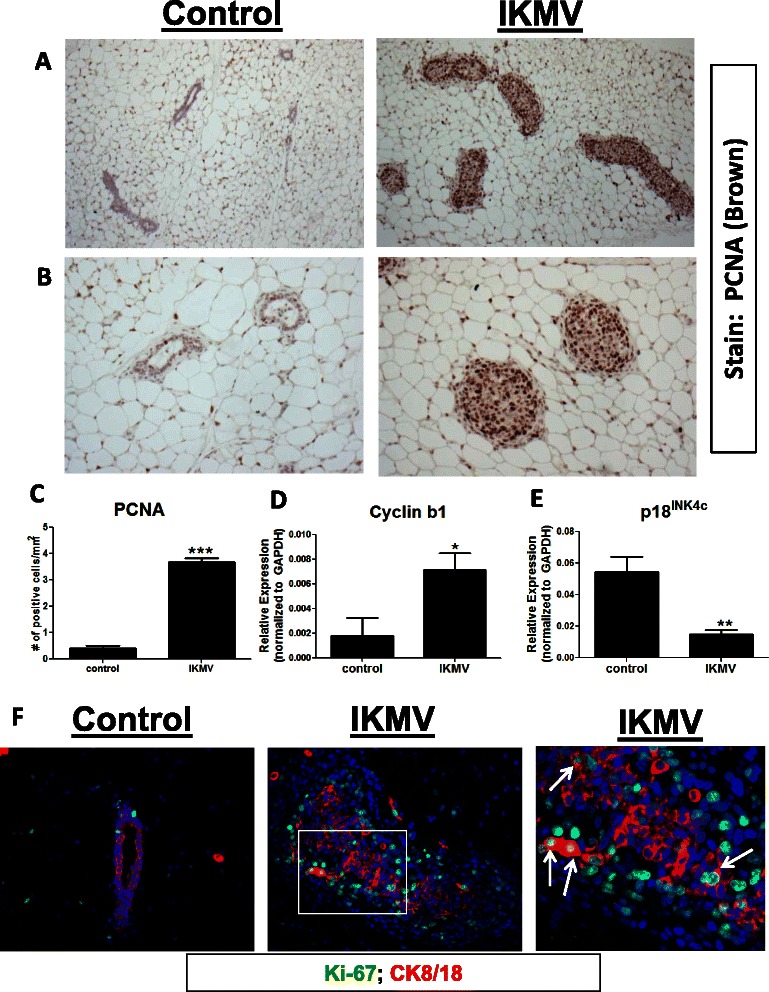
*Cells filling abnormal IKMV ducts are highly proliferative.* 6 week old virgin IKMV and control littermates were dox-treated (2 g/L) for 3 days prior to sacrifice. PCNA staining of mammary tissue reveals increased proliferation in the IKMV ducts as compared to controls, shown in both **a** 10X and **b** 20X images (positive staining is dark brown). **c** This increased number of PCNA positive cells/duct was quantified (counts were normalized to area of each duct; *n* = 3 control, *n* = 3 IKMV glands were used for quantification and 6 ducts per gland were counted; ***p < 0.0001). **d**,**e** qRT-PCR analysis shows increased expression of cyclin b1 and decreased expression of p18^INK4c^ following activation of NF-κB in mammary epithelium (*n* = 6 control, *n* = 7 IKMV samples run for cyclin b1; *p = 0.0210; *n* = 3 control, *n* = 4 IKMV samples run for p18^IKN4c^; *p = 0.0064). **f** Immunofluorescent staining for Ki-67 (green, nuclear) confirms that cells within the IKMV ducts are actively cycling. Many of the proliferating cells co-stain with CK8/18 (red, cytoplasmic), indicating that the luminal epithelium is driven to proliferate upon activation of NF-κB. Note that the far right image is a magnification of the white box in the center image. White arrows indicate double positive Ki-67/CK8/18 cells

Lumen-filling can be the result of decreased apoptosis and/or increased proliferation and NF-κB signaling plays a role in both of these cellular processes [26]. To determine the mechanism most relevant to the rapid filling of the IKMV ducts, we assessed the mammary tissue from 3 day dox treated IKMV and control mice to detect changes in apoptosis or proliferation. To quantify apoptotic cells, we stained the tissue with caspase-3, but found no significant change in the number of caspase-3 positive cells in IKMV vs. control tissue (data not shown). To assess proliferation in the glands, we stained for proliferating cell nuclear antigen (PCNA) (Fig. 6a-c). This revealed a profound increase in the number of proliferating cells within the IKMV ducts. All of the enlarged ductal structures contained PCNA positive cells, indicating that proliferation is the principle mechanism by which the ducts become filled with epithelium in such a short span of time.

Next, we looked for molecular markers of increased epithelial proliferation present in the IKMV glands. Cyclin b1is known to induce cellular transition from G2 to M phase and is often overexpressed in human breast malignancies [27]. Quantitative PCR (qRT-PCR) revealed increased mRNA expression of cyclin b1 in IKMV mammary tissue (Fig. 6d). Furthermore, expression of the mitotic inhibitor p18^INK4c^ (CDKN2C) was significantly decreased in IKMV tissue (Fig. 6e). This change is consistent with the observed phenotype, as p18^INK4c^ normally functions to restrain luminal progenitor cell expansion and inhibit luminal tumorigenesis in the mammary gland [28].

To further confirm that it was truly epithelial cells undergoing proliferation within the IKMV ducts, we co-stained control and IKMV mammary tissue (collected after 3 days of dox treatment) with the proliferative marker Ki-67 and the luminal epithelial marker CK8/18 (Fig. 6f). Ki-67 staining confirmed our earlier findings using the PCNA stain, as nuclear Ki-67 was observed in cells throughout the IKMV ducts. A number of these proliferating cells were also positive for CK8/18 (double positive cells indicated by arrows, Fig. 6f).

### Increased expression of inflammatory markers in IKMV mammary is concurrent with the down-regulation of hormone receptors and markers of epithelial differentiation

**Fig. 7:**
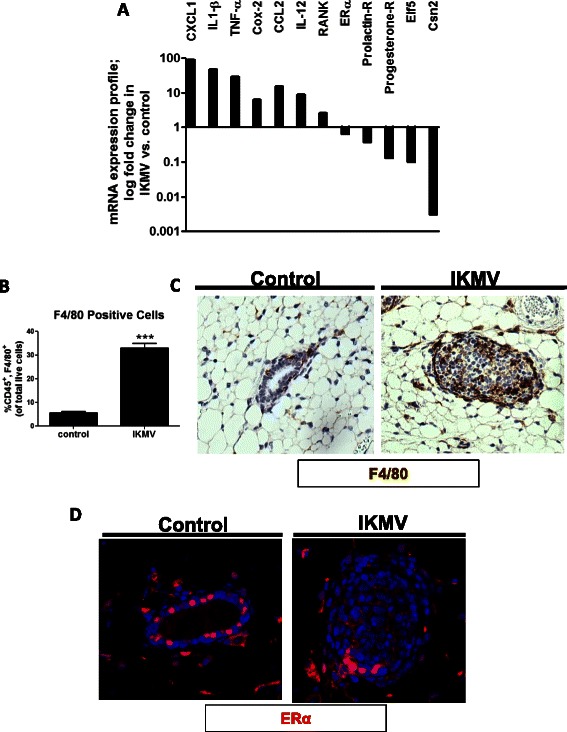
*Aberrant activation of NF-κB in mammary epithelium induces downstream signaling consistent with early tumorigenesis.* RNA was isolated from mammary glands of IKMV and control mice after 3 days of dox treatment (6 week time point). **a** qRT-PCR for a variety of gene targets revealed increased expression of inflammatory markers as well as decreased expression of hormone receptors and markers of epithelial differentiation (bars represent log fold change of IKMV vs. control; p values for each comparison can be found in Table 2. **b** Flow cytometry indicates there is an increased percentage of CD45^+^/F4/80^+^ macrophages in mammary glands following NF-κB activation, and **c** immunohistochemistry reveals that F4/80 positive macrophages have infiltrated the mammary ducts of IKMV mice (positive cells are dark brown). **d** Immunofluorescent staining reveals decreased nuclear localization of ERα in IKMV ducts (red; dense and nuclear in control epithelium)

Our results show that activation of NF-κB in mammary epithelium results in a rapid proliferative response and induces a hyperplastic ductal structure. Because NF-κB signaling can drive downstream expression (and sometimes repression) of hundreds of gene targets, we wondered what other molecular changes were occurring after NF-κB activation in the mammary ducts and how they could be mediating the observed phenotype. To achieve this, we completed a panel of qRT-PCR assays (Fig. 7a). Our analysis revealed that downstream read-outs of inflammatory signaling, such as CXCL1, IL-1β, TNF-α, and Cox-2, were significantly increased in the IKMV tissue. Interestingly, increased expression of many of these products (IL-1β, TNFα, and Cox-2) has been implicated in previous studies to contribute to mammary hyperplasia and tumorigenesis [29–31]. In addition, our analysis revealed a small but significant increase in the expression of Receptor Activator of NF-κB Kinase (RANK) in the IKMV mammary gland. Previous studies have shown that increased signaling through RANK/RANK-L in the mammary gland can lead to expansion of the epithelium, disruptions in differentiation, and disorganization of the basal/luminal structure. Also up-regulated were genes associated with an influx of inflammatory cells, specifically macrophages. CCL2 is a macrophage chemoattractant, and its expression was increased in the activated tissue. Accordingly, we found an increased percentage of F4/80 positive cells within the IKMV mammary tissue via flow cytometry analysis (Fig. 7b). Immunohistochemistry for F4/80 revealed that in control samples, macrophages are sparse and localized along the outer edge of the ducts, as expected. In the IKMV tissue, the F4/80 positive cells appear both around the outer edge and on the inside of the ducts, interspersed between the hyperplastic epithelium (Fig. 7c). We also detected an increased level of IL-12 transcript in the IKMV tissue, indicating that the recruited macrophages are likely more aligned with a classically activated phenotype.

Converse to the up-regulation of these inflammatory components, we observed decreased hormone receptor expression in the IKMV mammary, including lower levels of estrogen receptor alpha (ERα), progesterone receptor, and prolactin receptor. Immunofluorescent staining reveals decreased nuclear localization of ERα in IKMV ducts (Fig. 7d). These decreases suggest that the proliferative response cannot be explained by increases in hormonal signaling. We also observed a striking decrease in the expression of β-casein (Csn2), a marker of mammary epithelial differentiation. In addition, expression of Elf-5, a key regulator of mammary epithelial differentiation, was also decreased in IKMV epithelium. Deletion of Elf-5 can lead to disorganized mammary structures and collapsed lumens, suggesting that this decrease in expression could be playing a role in the observed phenotype [32].

### Pathological review of IKMV tissue confirms diagnosis of low-grade DCIS

To determine the staging of the IKMV ductal abnormalities, the H&E stained sections were analyzed by Dr. Thomas Stricker MD/PhD, pathologist at Vanderbilt University Medical Center. Dr. Striker observed that the ducts in the IKMV mice were expanded, containing mildly atypical cells with increased nuclear-cytoplasmic ratio, chromatin condensation, nuclear membrane abnormalities, and nucleoli. Furthermore, he found that cells in the glands were well spaced out with few overlapping nuclei and that there were increased numbers of apoptotic cells. Taken together, he concluded that these findings are consistent with a diagnosis of low-grade DCIS.

### MUC1 oncoprotein is up-regulated and mislocalized in IKMV mammary epithelium

**Fig. 8:**
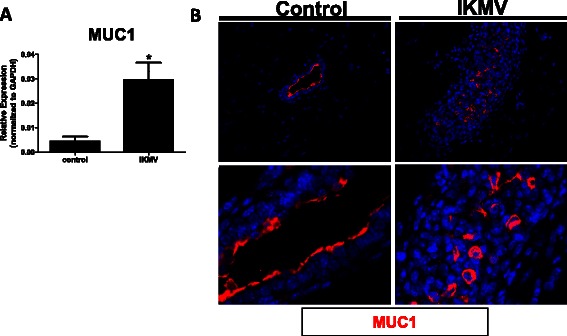
*MUC1 oncoprotein is up-regulated and mislocalized in IKMV mammary epithelium.* RNA was isolated from mammary glands of IKMV and control mice after 3 days of dox treatment (6 week time point). **a** The hyperplastic IKMV ducts have increased expression of MUC1 via qRT-PCR (*n* = 5 control, *n* = 6 IKMV samples; *p = 0.0119). **b** Staining for MUC1 indicates that it is properly localized to the apical surface of luminal epithelium in control ducts but it has become repositioned to the entire cell membrane in many of the cells within the IKMV hyperplastic ducts (red staining is MUC1, blue is DAPI; 40X images are shown; images at bottom are magnified to show detail)

To further strengthen correlations between the IKMV phenotype and DCIS, we analyzed the expression and localization of Mucin 1 (MUC1). This protein is normally expressed on the apical surface of mammary epithelium where, similar to other mucins, it plays a role in host defense against pathogens. Beyond this role in normal physiology, MUC1 is considered an oncoprotein, as its overexpression can functionally drive malignant transformation in breast epithelium [33]. When luminal epithelial cells lose their polarity due to stress or transformation, MUC1 can be expressed around the entire cell membrane rather than staying localized to the apical surface. This repositioning of MUC1 has been noted in both ductal hyperplasia with atypia and in DCIS of the breast [34]. We analyzed our 6 week virgin, 3 day dox treated samples for MUC1 expression and observed a significant up-regulation of MUC1 in IKMV tissue via qRT-PCR (Fig. 8a). Further, we completed immunofluorescent staining and found that IKMV ducts contained MUC1 positive cells dispersed throughout the filled lumens (Fig. 8b). These positive cells displayed MUC1 staining around the entire cell membrane. This was in contrast to the control ducts, which had appropriately localized MUC1 along the apical surface of each lumen.

## Discussion

In this study, we have modeled specific activation of NF-κB signaling in virgin mammary epithelium and demonstrated a variety of downstream morphological and molecular consequences.

Transplant studies using IKMV transgenic tissue revealed that aberrant activation of NF-κB during ductal outgrowth leads to hyper-branched, hyperplastic ductal structures. The resulting phenotype is strikingly similar to the studies completed using IκBα KO tissue even though the current model uses a different means of pathway intervention (constitutive IKKβ). In addition, further analyses in both models indicated that the expanded epithelium in activated tissue was the result of increased proliferation, not decreased apoptosis. Because the IKMV transgene is epithelial specific, we definitively show that aberrant NF-κB signaling originating within the epithelium can drive these morphological changes. It has been suggested that hyperplastic growth of ductal tissue is the result of stromal changes and that the epithelium is influenced to become malignant because its environment has provided a permissive niche [35]. In future studies, it would be intriguing to determine if the observed alterations in epithelial signaling and structure could be induced by starting the cross-talk from the other direction and initiating NF-κB activation specifically in the stroma. One published study suggests this may be the case: when AEBP1, an inflammatory mediator, was overexpressed in macrophages and adipocytes, it was able to activate NF-κB activity in the mammary gland and led to alveolar hyperplasia [36].

After completing the transplant studies, we hypothesized that NF-κB-driven hyperplastic growth might play a role in the formation of hyperplastic breast lesions, or DCIS. This could now be formally tested using the temporal control provided in the IKMV model. We found that we could establish a network of normal ducts in 6 week old virgin females, and then induce hyperplasia through 3 days of aberrant NF-κB signaling. In addition, similar changes occurred upon activation of the epithelium in a fully adult, 16 week old gland. One striking aspect of this phenotype is its rapid induction. Though aberrant signaling through other cell-signaling pathways is certainly able to induce mammary ductal hyperplasia, it is often over a period of weeks or months, not days. A noted exception is activation of FGF-receptor 1 (FGFR-1) in mammary epithelium. Welm et al. induced FGFR-1 transgene activation at the 6 week virgin time point and observed rapid formation of hyperplastic ductal structures accompanied by increased inflammatory signaling and macrophage recruitment [37]. It was later shown that IL1-β secretion by the recruited macrophages was playing a significant role in the hyperplastic growth, as blocking IL1-β or depleting macrophages abrogated the effects of FGFR-1 signaling [29]. They note that IL1-β treatment of mammary epithelial cells induces NF-κB activation, which suggests that the phenotype we observe in the IKMV model could be mediated, at least in part, by paracrine IL1-β secretion. While the timing is similar, hyperplastic growth in the FGFR-1 model is the result of increased lateral budding of the epithelium, whereas the IKMV model exhibits lumen-filling and duct enlargement. Thus, there are likely somewhat different mechanisms at play in the two systems.

Another striking feature of the IKMV short-term model is the robust proliferative response induced after NF-κB activation. Mechanistically, this could be mediated by direct binding of NF-κB to the promoter of cell-cycle mediators, as it has been shown to transcriptionally regulate cyclin d1[38]. However, we did not find a significant increase in cyclin d1 mRNA expression in IKMV vs. control glands after the three day dox treatment (data not shown). We did observe changes in two other cell cycle mediators: cyclin b1 and p18^INK4c^. As noted earlier, both an increase in cyclin b1 and a decrease in p18^INK4c^ have been associated with pro-tumorigenic changes in the breast. No study has noted the presence of an NF-κB consensus binding site in either of these genes’ promoters. Nevertheless, some reports indicate that p65 (and other family members) can bind to numerous sites other than the recognized consensus sequence, such as Alu-repetitive elements in DNA. Via CHIP analysis, it was shown that NF-κB bound the p18^INK4c^ promoter, which contains these Alu-repeats, following viral infection of Hela cells [39]. Continued studies of how NF-κB can directly regulate expression of numerous cell cycle related genes is warranted, given these findings. In addition, proliferation in the IKMV ducts could be induced through an indirect mechanism, mediated by NF-κB-driven production of pro-inflammatory factors. Increased expression of both TNF-α and Cox-2 was apparent in the IKMV tissue. TNF-α has been shown to promote anchorage independent growth and invasion in mammary epithelial cells, and overexpression of Cox-2 in mammary epithelium is sufficient to induce hyperplastic growth of virgin ducts [30, 31]. Likely, a combination of both direct and indirect mechanisms leads to proliferation and hyperplastic growth in the IKMV ducts.

We determined that aberrant NF-κB activation leads to decreased expression of hormone receptors and other markers of mammary epithelial differentiation such as Elf5 and CSN2. This is consistent with our previous report of a dramatic decrease in CSN2 mRNA expression and protein levels upon NF-κB activation in lactating mammary glands [12]. It was also previously shown that constitutive activation of RANK in the mammary epithelium can lead to decreased ELF5 expression [40]. These observed decreases in markers of epithelial differentiation following NF-κB activation are consistent with reports that NF-κB can function to reprogram mammary epithelium, leading to epithelial to mesenchymal transition (EMT) [41]. We did not detect significant mRNA changes in classic indicators of EMT such as Vimentin, Zo-1, E-cadherin, or N-cadherin, in the IKMV tissue (data not shown), but it is possible that these types of changes may be more apparent at other time points.

We observed two additional clinical markers of tumorigenesis within the hyperplastic IKMV ducts: infiltration of macrophages and the up-regulation and mislocalization of MUC1. Macrophage infiltration and CCL2 expression are correlated with poor prognosis and metastasis in human breast cancer [42, 43]. It has been shown that NF-κB activation in the mammary epithelium enhances macrophage recruitment to the site of primary mammary tumors in both the polyoma middle T and Erb2 oncogene-driven mouse mammary tumor models [16, 18]. In the current study, we find that NF-κB activation, in the absence of any oncogenic mutation, results in significant macrophage infiltration into the ducts. These immune cells are likely acting in an “M1” role initially, responding to the inflammatory signals as they would to an infection. It will be interesting in future studies to parse the individual sub-classes of macrophages that are recruited to the mammary ducts and determine whether their phenotype may become more pro-tumor, or “M2”, with time.

MUC1 expression is a topic of great interest in a variety of cancer types, and particularly in breast. It is overexpressed in 90 % of human breast cancers, and the secreted form can be detected in the serum of many patients, even those with non-metastatic disease [44]. Thus, it is actively being pursued as a serum bio-marker for early detection. In a previous study, overexpression of the MUC1 cytoplasmic domain in mouse mammary tissue resulted in hyper-branched, hyperplastic ducts with an increased number of terminal end buds [45]. Activation of NF-κB is one downstream effect of MUC1 signaling, and may have been contributing to the hyperplastic phenotype of that model. In addition, it has been suggested that MUC1 and p65 participate in an auto-inductive loop, as each has been shown to increase expression and/or signaling of the other [33]. Once MUC1 becomes overexpressed and repositioned along the entire cell membrane, it can activate a number of receptor tyrosine kinases, most notably epidermal growth factor receptor (EGFR) [46]. It can also bind to beta-catenin and play a role in activating its downstream target gene targets [45]. Whether MUC1 signaling plays a critical role in the IKMV phenotype remains to be determined. Inhibitors of MUC1 are being developed, which could be combined with our model in future studies to answer this intriguing question [47, 48].

Upon pathological review, we found that the IKMV lesions meet the criteria for the diagnosis of low-grade DCIS [49]. The rate of diagnosis of DCIS in American women has increased due to mammography, and it is currently being debated how aggressively these lesions should be treated. Some argue for minimal treatment, citing that DCIS has been found at autopsy, and thus may never progress to a life-threatening condition in a subset of women [50]. However, one retrospective study estimated that 28 % of women treated with biopsy-only for DCIS will develop invasive carcinoma in a follow-up period of approximately 15 years, suggesting that more aggressive treatment is warranted [51]. In the absence of definitive markers of whether the disease will progress beyond DCIS, the frequently suggested treatment is mastectomy or lumpectomy followed by radiation therapy. This strategy appears highly effective with an extremely high percentage of such patients surviving ten years later. However, these women have then undergone the same aggressive treatment as would be proposed for invasive disease. It remains unclear what additional factors may lead the contained lesions into becoming invasive and metastatic as opposed to remaining as DCIS. This is a critical area for research, and future studies using the IKMV model could yield important insights into what physiological and environmental factors combine with inflammatory signaling to promote malignancy in the breast.

## Conclusion

Our model underscores the previously unappreciated effects of short term, aberrant activation of NF-κB signaling in developmentally normal mammary epithelium. While prolonged inflammatory signaling is recognized as a risk factor for tumorigenesis, we now show that even a short pulse of NF-κB hyper-activation can lead to pre-malignant changes in the breast. Similar changes in architecture and molecular signaling could be occurring in human breast tissue after acute infection, injury, or stress. Thus, inhibition of NF-κB signaling following acute inflammation or the initial signs of hyperplastic growth could represent an important opportunity for breast cancer prevention.

## Additional file

Additional file 1: **Gating strategy for FLOW cytometry data in Fig.** **7b****.** An average of 100,000 events were counted for each sample. To start, all samples were taken through the first three gates (top, labeled 1, 2, 3), which excluded artifacts that were not single-cells based on forward and side scatter. From there, DAPI stain was used to determine viability (gate 4). All DAPI negative cells were carried to gate 5, where cells were split into CD45 positive and CD45 negative populations. The CD45 positive population was then gated using F4/80 on the x axis and CD45 on the y axis (gate 6). Circles indicate CD45 + F4/80+ cells. Values for the graph in Fig. 7b were obtained by taking the total number of CD45^+^F4/80^+^ cells counted for each sample and dividing that value by the total number of viable cells counted in the sample (DAPI negative). (PDF 309 kb)

## Abbreviations

AEBP1: Adipocyte Enhancer-Binding Protein 1
CCL2: Chemokine (C-C motif) ligand 2
MCP-1: Monocyte chemotactic protein 1
CHIP: Chromatin immunoprecipitation
cIKKβ: Constitutive inhibitor of kappa B kinase beta
CK5: Cytokeratin 5
CK8/18: Cytokeratin 8/18
Cox-2: Cyclooxygenase 2
Csn2: beta casein 2
DCIS: Ductal carcinoma in situ
dox: doxycycline
EGFR: Epidermal growth factor receptor
EMT: Epithelial to mesenchymal transition
ERα: Estrogen receptor alpha
FGFR-1: Fibroblast growth factor receptor 1
IKKβ: Inhibitor of kappa B kinase beta
IKMV: double transgenic mouse model used in these studies which allows activation of nuclear factor kappa B signaling specifically in mammary epithelium upon addition of doxycycline to mouse drinking water
IL-1β: Interleukin 1 beta
IκBα: Inhibitor of kappa B alpha
KO: Knock out
MMTV-rtTA: Mouse mammary tumor virus reverse tetracycline transactivator
MUC1: Mucin 1
NF-κB: Nuclear factor kappa B
PCNA: Proliferating cell nuclear antigen
qRT-PCR: quantitative reverse transcription polymerase chain reaction
RANK: Receptor Activator of NF-κB Kinase
SMA: Smooth muscle actin
TEB: Terminal end bud
