# Supplementary figures and images for: Aberrant activation of NF-κB signaling in mammary epithelium leads to abnormal growth and ductal carcinoma in situ
Source: BMC Cancer. 2015 Sep 30;15:647. doi: 10.1186/s12885-015-1652-8 (PMC4590702; doi:10.1186/s12885-015-1652-8)

## Additional File 1.

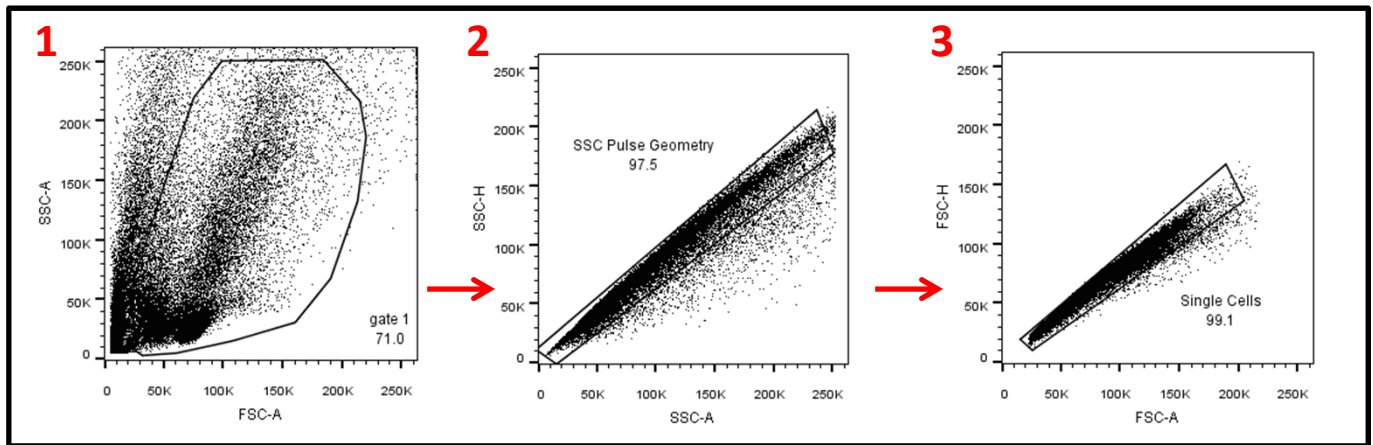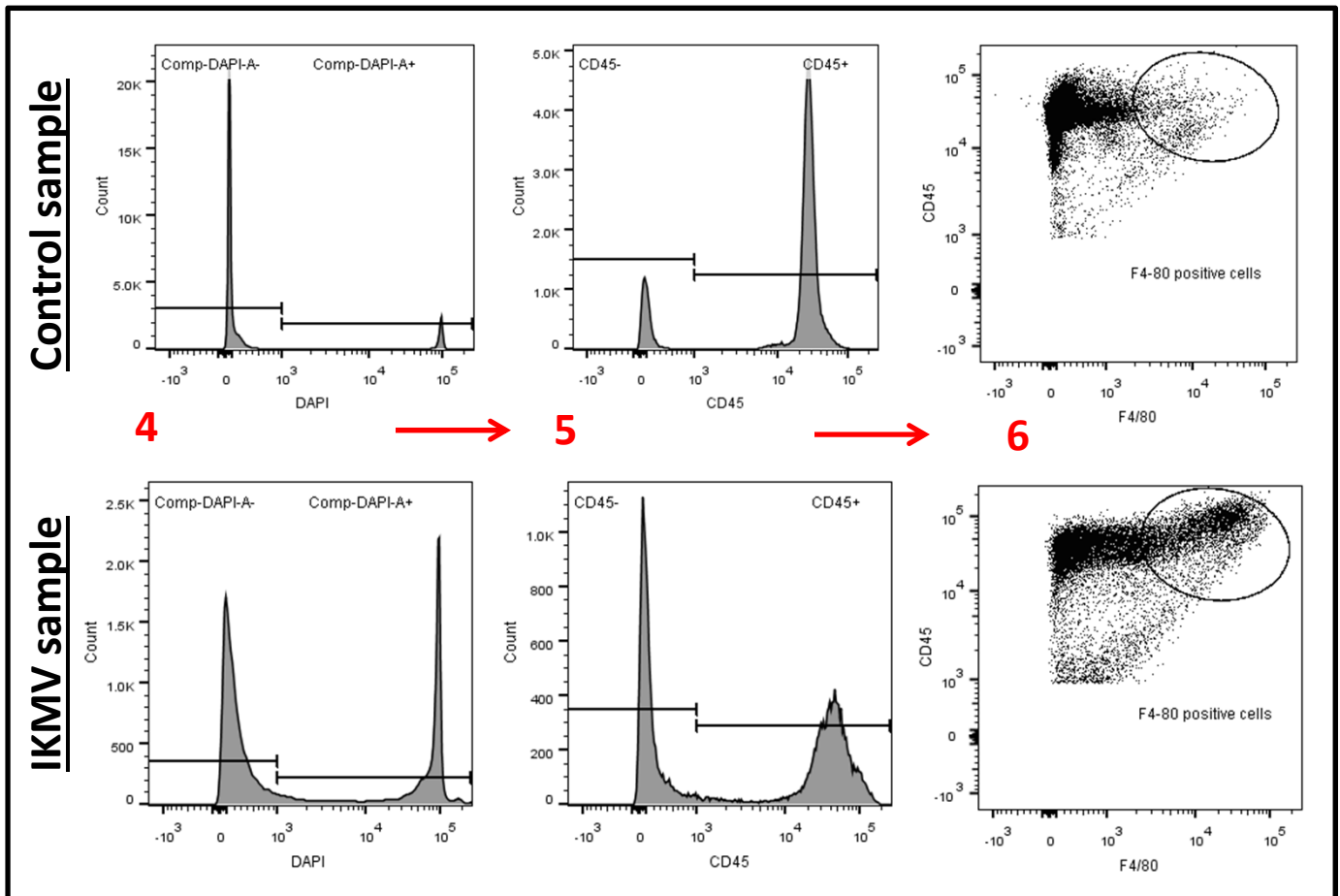

Supplement: Additional file 1: — Gating strategy for FLOW cytometry data in Fig. 7b. An average of 100,000 events were counted for each sample. To start, all samples were taken through the first three gates (top, labeled 1, 2, 3), which excluded artifacts that were not single-cells based on forward and side scatter. From there, DAPI stain was used to determine viability (gate 4). All DAPI negative cells were carried to gate 5, where cells were split into CD45 positive and CD45 negative populations. The CD45 positive population was then gated using F4/80 on the x axis and CD45 on the y axis (gate 6). Circles indicate CD45 + F4/80+ cells. Values for the graph in Fig. 7b were obtained by taking the total number of CD45+F4/80+ cells counted for each sample and dividing that value by the total number of viable cells counted in the sample (DAPI negative). (PDF 309 kb) [file 12885_2015_1652_MOESM1_ESM.pdf]
